# Impact of glycogen resynthesis on lean mass

**DOI:** 10.1186/1550-2783-11-S1-P43

**Published:** 2014-12-01

**Authors:** Jacob A Ormes, Ryan P Lowery, Jeremy E Silva, Jacob T Rauch, Sean A McCleary, Matthew H Sharp, Kevin A Shields, John I Georges, Jacob M Wilson

**Affiliations:** 1 The University of Tampa, Tampa, Florida, USA

## Background

It has frequently been demonstrated that resistance training has a negative effect on muscle glycogen content. Additionally, the rate of resynthesis seems to be dependent upon the degree of depletion. However, the impact of glycogen resynthesis on lean mass in a resistance trained population consuming a very low carbohydrate diet has yet to be examined. This has important implications for athletic populations as body composition appears to be related to performance]. Therefore, the purpose of this study was to examine the effects of glycogen resynthesis on body composition in resistance trained individuals consuming a ketogenic diet.

## Methods

Thirteen experienced resistance trained males volunteered to participate in this study (mean ± SD, age: 23.5 ± 3.3, weight: 187.6 ± 32.6) and were instructed to consume a ketogenic diet consisting of 5% carbohydrate, 25% protein, and 70% fat for eight weeks. Additionally, subjects were engaged in a monitored, periodized resistance training program for the duration of the study. On week nine, carbohydrates were gradually reintroduced to the diet at a rate of 1g/kg. This rate increased by 1g/kg at two day intervals for a total of 3/kg throughout the week. Body composition (Hologic Dual X-Ray Absorptiometry) and ultrasonography determined muscle thickness were measured at Week 0, 8, and 9. Consent to publish the results was obtained from all participants.

## Results

Total Mass, LBM, and quadriceps thickness significantly increased (p < .05) from week 8 to week 9 by 4.81 kg ± 2.8, 2.9 kg ± 2.1, and 0.2 ± 0.2 cm, respectively, meanwhile fat mass significantly decreased by 1.8 kg ± 1.3.

## Conclusion

The primary finding of this study is that the reintroduction of carbohydrate for one week in a depleted population significantly increases DXA determined lean body mass.

